# ‘No‐one has listened to anything I’ve got to say before’: Co‐design with people who are sleeping rough

**DOI:** 10.1111/hex.13235

**Published:** 2021-03-23

**Authors:** Robyn M. Mullins, Bridget E. Kelly, Patrick ‘Spike’ Chiappalone, Virginia J. Lewis

**Affiliations:** ^1^ Australian Institute for Primary Care and Ageing La Trobe University Bundoora Victoria Australia; ^2^ cohealth Melbourne Victoria Australia

**Keywords:** Australia, co‐design, homeless persons, housing, qualitative, social problems

## Abstract

**Background:**

Despite policies and programmes aimed at housing people who are homeless, there are still people who live and sleep rough. This project used the skills and knowledge of people in this situation to identify a strategy to mitigate some of the risks.

**Objective:**

To describe the development and conduct of a co‐design project involving people who are homeless.

**Setting/Group Members:**

A Working Group of 11 was formed following a careful recruitment process from people who had volunteered after consultation by the project team. The co‐design approach was guided by a set of principles.

**Methods:**

Eight members of the Working Group were interviewed by an external researcher (RM). The approach was primarily deductive, with the principles adopted by the project team used as a framework for data collection and analysis. The co‐design process was captured by the project leaders (BK, PC) supplemented with documentation review and team discussions.

**Results:**

The group met weekly for 12 weeks, with 8‐10 members present on average. They reviewed information from the survey, contributed ideas for solutions and ultimately decided to provide information via print, a website and an event. Important factors in on‐going involvement were carefully selecting group members and making participation rewarding for them.

**Discussion/Conclusions:**

Vulnerable people such as those experiencing homelessness can be excluded from decision‐making processes affecting them, as they can be perceived as hard to reach and unable to make a meaningful contribution. This project demonstrated that a carefully managed project, with sufficient resources and commitment, it was possible to involve people who are homeless and maintain involvement over an extended time period.

**Public Contribution:**

The Working Group reviewed survey findings and developed an intervention to minimize the health, social and legal harms of sleeping rough. Several members reviewe this paper.

## 1 INTRODUCTION

The recognition that consumers have a valuable contribution to make in the way health and social services are delivered acknowledges that lived experience is a form of expertise which can complement professional expertise.^1^ Co‐design is based in long‐standing practices such as community engagement and public participation, as well as patient engagement.

There is no agreed definition of co‐design, and ‘when co‐design is loosely defined or operationalized as any type of collaborative or participatory activity, almost everyone seems to be doing it’.^2^ It has been argued that co‐design and similar terms such as co‐planning and co‐production are actually just new terms for the long‐existing practices in health promotion of partnerships and collaboration.^3^ What is generally agreed is that as a form of engagement co‐design goes beyond seeking feedback from target groups and end‐users in formats such as focus groups or surveys to ‘high‐level engagement… [which] represents a partnership in the design or evaluation of services’.^4^ In essence, co‐design recognizes that ‘different perspectives and a productive combination of different perspectives’ are needed to understand the needs of users and develop successful services.^5^

People from lower socio‐economic groups are known to be under‐represented in all forms of health and medical research.^6^ As co‐design, and other variations of participant engagement, has become more widely adopted, concerns have been raised about the way disadvantaged or vulnerable populations may not be appropriately involved, and that participants often comprise convenience samples resulting in tokenistic participation.^5^, ^7^ Reported barriers to the inclusion of vulnerable groups include issues with initial engagement; power differentials; and health, economic and social circumstances affecting participation.^8^ Vulnerable people may also lack some of the skills which are needed to participate fully in existing processes, such as communication and general cognitive skills, as well as willingness to become part of groups in the first place—which may in turn lead to increased marginalization as more vocal groups have their voices heard.^9^ For people in the homeless community, social stigma has a demoralizing impact which can inhibit them from coming forward to seek services and health care; it can also be a barrier to participating in consultation and engagement when opportunities do arise.

Some of the most vulnerable and disadvantaged people in Australia are those who are sleeping rough. Although there are some successful programmes aimed at providing housing for those who need it,^8^, ^10^ funding and housing stock is inadequate, and some of the people who are still excluded from housing are those with the most complex needs.^11^ In 2016, it was estimated that approximately 1000 Victorians were sleeping rough.^12^ Using co‐design with people with lived experience of homelessness to develop strategies that may improve health and well‐being is required because of the specific nature of the knowledge and the negative consequences of lack of information.

Even in high‐income countries such as Australia, people who are homeless have been shown to have high rates of morbidity and mortality, including substance use issues, communicable and non‐communicable diseases, and psychiatric problems.^12^, ^13^ They also tend to seek care late, once health issues become emergencies, in part because the efforts involved in meeting basic needs such as food, water and shelter take priority.^14^ Despite recognition of the barriers faced by this group, we could not find any examples of co‐design undertaken over a period of months with people who are sleeping rough in the refereed literature, as opposed to one‐off consultations or co‐design not directly involving the homeless.^15^, ^16^

The goal of this paper was to evaluate a project that used co‐design principles to utilize the skills, knowledge and experiences of people with a current or recent experience of rough sleeping to identify some of the health, social and legal issues faced when sleeping rough and strategies to address them.

## 2 OVERVIEW OF THE PROJECT AND RESEARCH

The project was initiated by cohealth which is one of Australia's largest community health services, which has a mission to improve health and well‐being for all, and to tackle inequality and health inequity in partnership with people and their communities.

A two‐person team was appointed to manage the project, a Project Worker (BK) with a social work background and a Peer Worker (PC) who has a lived experience of homelessness, is a graduate of the Council to Homeless Persons (CHP) Peer Education Support Program and who was employed at cohealth in an Allied Health Assistant role. BK and PC are also co‐authors on this paper.

The background work prior to the co‐design project is not reported in detail in this paper, but in brief, it comprised developing and collecting information using a survey which was completed by 81 people with current or recent experience of sleeping rough.^17^ Cohealth's internal Human Ethics Advisory Group approved the project (HEAG 1902), consistent with the organization's policies for internally initiated data collection without the intent to publish.

After the consultation data were collected, a Working Group was put together to review the survey findings, identify the key issues faced by people sleeping rough and consider strategies to support people to stay healthy and safe. The group met weekly for 12 weeks from September to December 2019, facilitated by BK and PC. PowerPoint slides were used to structure the sessions. The format of each working group meeting was:

- Acknowledgement of Country (a statement that shows awareness of and respect for the Traditional Custodians of the land).
- Reminder about the group agreement (see below).
- Start‐off activity such as poems, mindfulness exercises.
- Updates including things such as other opportunities for involvement, current campaigns and things happening within homelessness, as well as information about things the facilitators had been doing between the workshops.
- Reviewing the findings from the survey, with discussion and reflection based on presentations made using graphs, quotes and thematically analysed data.
- Activities including small group work and large group work, for example matching aims to proposed ideas, matching findings from the survey to ideas about how to respond, developing an idea prioritization matrix, narrative activities and storyboarding.
- Closing comments, including ‘what's on next time’ and at key timepoints, evaluation questions.

Within this overall structure, each session had a specific focus:

**Table:** 

Session 1:	Overview: Background to the project
	Explanation of funding available, and approval pathways which need to be followed outside of group
	Explanation that a working group is time‐limited, and has a task to complete
	Discussion and signing position description/consent form
	Developing the group agreement on conduct
Sessions 2‐8:	Predominantly discussion of results from the survey and ideas for addressing the issues:
	2. Safety
	3. Health and basic needs
	4. Public Space/Doing things
	5. Council Laws, Belongings, Storage
	6. Legal Issues
	7. Services and Money
	8. ID and Communication
Sessions 9‐12:	Predominantly designing the resource the group had agreed to produce:
	9. Refocussing on the aims, discussing
	10. What is the best way to have an impact? What ideas can be brought into a resource? Who are we targeting?
	11. Refining ideas (high or low impact/easy or hard to achieve)
	12. Agreement on producing something to share information peer‐to‐peer, and storyboarding how it would be put together

Some core guiding co‐design principles were adopted by the programme staff when developing this project. These were based on literature^18^, ^19^ particularly principles from the Co‐design Initiative, as well as personal experience:

- Inclusion: recruiting a diverse group of people with a recent lived experience of rough sleeping.
- Equity (and respect): ensuring that all voices in the group were heard and valued.
- Capacity building: supporting people to participate safely, and in a way that is empowering.
- Purposeful: working towards a useful outcome.

Other key principles from the Co‐design Initiative of ‘co‐created’, ‘innovative’ and ‘evaluated’ relate to the product which resulted from the co‐design process, which is out of scope for this paper.

The final outcome was a zine (an informal magazine, self‐published and frequently produced on a photocopier) which was made available in print and online (http://www.needtoknowhomeless.org.au/) as well as a website and an event where information could be read out. It contained information, stories, encouragement and advice that could help people who were sleeping rough, for example advice for staying healthy on the streets. Subject to funding, the plan is to produce it regularly.

During the co‐design process, independent university researchers were asked to evaluate some aspects of the project, specifically the way the Working Group had been put together and run. They conducted interviews with Working Group members, reviewed documents and held discussions with the project staff. The evaluation was approved by La Trobe University's Low Risk Human Ethics Committee (HEC20182). Evaluation of the product (the zine) was not the focus.

The objectives of this paper are to describe:

1. The process of recruiting and maintaining involvement over time of people who were sleeping rough in a Working Group to co‐design support for them and their peers.
2. The views of the members of the Working Group about their participation.
3. The views of the facilitators about the co‐design process.

## 3 EVALUATION METHODS

### 3.1 Interviews with the group members

In June 2020, all members of the Working Group were contacted and asked to take part in an interview. Three of the original members were no longer actively participating and all of these declined to be interviewed. Eight interviews were conducted: five face‐to‐face and three by telephone. The face‐to‐face interviews were conducted at cohealth, next door to where the Working Group meetings had been held, to ensure the interviewees were as comfortable as possible. Written consent was obtained at face‐to‐face interviews, and verbal consent was recorded for telephone interviews.

The four co‐design principles used to develop the project formed a framework for the semi‐structured interview schedule. The questions focussed on the group members’ motivation to be part of the Working Group, their experiences of being selected and then being part of the group, things they felt could be changed about the overall process, and their satisfaction with the zine (Appendix A).

Interviews were audio‐recorded and then played back while detailed notes were taken. The analysis approach was primarily deductive, guided by the co‐design principles that provided the framework for the project's development. The analysis method followed the general approach suggested by Braun and Clark^20^ with notes read over frequently for familiarization and initial identification of patterns (RM). Themes were reviewed and confirmed through discussions between RM and VL. BK and PC were not involved in the analysis of the interviews, as they would have easily been able to link the interview to a specific group member. Group members were assured their interviews were confidential, and all quotations use gender neutral pseudonyms, to help protect their privacy. Quotations included in this paper use ellipses to remove irrelevant asides and unnecessary words,^21^ but are otherwise verbatim.

### 3.2 Other input

Documentation collected throughout the project was reviewed, including the PowerPoint presentation used in each session. Additionally, the facilitators have contributed their perspectives and are authors on this paper (BK, PC).

## 4 RESULTS

### 4.1 Inclusion

#### 4.1.1 Recruitment to the group

Initial interest in being part of the Working Group was gauged from those who completed a survey as part of the project's initial consultation of people experiencing homelessness. At the end of the survey, respondents were asked if they were interested in participating in a working group which would ‘discuss the responses to the survey and design and develop something that aims to reduce the health, legal and social impacts of sleeping rough’. They were advised that this would involve committing to 12 weekly 2‐hour group sessions for which they would be paid. Sixty‐one of 81 people surveyed expressed interest at the time they were surveyed. A multi‐step process was then undertaken to select members of the Working Group.

1. Contact was made with those who had expressed interest in participation, either by telephone, email or via information to workers or services to pass on. At this point, people were given more information about the project (eg the weekly commitment needed).
2. A more formal telephone interview was conducted with potential candidates who were paid for their time. This covered their motivation to be part of the group, skills and experiences they would bring, their ability to get on with others and to self‐regulate their emotions. Availability to attend and support needed to do so (eg public transport cards) were also assessed.
3. The information from the interviews was discussed by BK and PC to develop a short‐list of potential members. This took into consideration socio‐demographic factors such as gender, age, ethnicity, LGBTQI and Indigenous backgrounds, length of time homeless/rough sleeping as well as the interest and capacity to participate identified in the telephone interviews. They were also asked how comfortable they would be discussing their own lived experience in the group.
4. The key candidates were interviewed face‐to‐face and final decisions were made based on consideration of the mix of personalities, how well they would work together, and how they could be expected to handle the experience of discussing potentially distressing content.

The initial group included seven men, four women and one non‐binary person; four were born overseas with two culturally and linguistically diverse; four identified as LGBTQI and one as Indigenous. They ranged in age and rough sleeping/homelessness experiences. One demographic which was not successfully recruited, despite extensive efforts, was young people.

#### 4.1.2 Inclusion: Joining and staying in the group

The Working Group members described their motivations for joining the Working Group primarily as the desire to share their insights and to help others who found themselves on the streets. A couple described feeling overwhelmed and happy when it was confirmed they would be on the Working Group. Although altruism was key, the financial incentives and other support offered also contributed to motivation. Some group members were clear that they would have participated even if they were not reimbursed for their time, though others felt it was appropriate that they were paid for their time and their skills and said that it also increased their sense of what they were doing as being their ‘work’. The provision of lunch and transport cards was welcomed, but more as part of feeling respected and valued than a key reason for being here.

Attendance at each group usually ranged from 8 to 10 people. At the time of the interviews, approximately 9 months after the first meeting, eight people remained actively involved. The eight who were interviewed had maintained their enthusiasm or become more motivated.

[I] probably got more motivated, because I was excited to see friends that I’d made, have that chat, have that discussion…towards the end it was more exciting to go knowing the environment I was going to be in. Jamie

It’s not important, but what I liked was the welcoming when you got there, when everyone got there. Everyone was nice, like hello to everyone. For me I don’t usually get that… Everyone was genuine, not being nice cos they wanted to get credit off you. Charlie

The facilitators identified some practical aspects of the meetings which made them enjoyable for the group members: tea, coffee, a nutritious lunch and snacks were supplied, as well as a selection of fidget toys (stress balls, Rubik's cubes) and weighted blankets and cushions which group members could access at any time. One group member remarked to a facilitator that it was a highlight just to be in a warm room with food.

### 4.2 Equity and respect: ensuring that all voices in the group were heard and valued

There was a strong sense of being respected and made comfortable by the facilitators and other staff at the community health service where they met.

Way we were treated…as equals. I think you expect to walk into a place like this, they’re professionals, this is a professional place, we’ve come in off the street. Expect them to treat ya like shit as per usual, like in shops or other places around…[But they] class us as workers here. Sam

The way Bridge and Spike facilitated it, wasn’t being run by them… it run by us as people with lived experience. They were there to bring us back to what we were supposed to be doing. Alex

The group members all felt they had their voices heard in this group, from one with more confidence who said ‘Mine more than anyone’ (Chris), to another self‐described ‘not much of a talker’ who said ‘I’m quite surprised but I do [think my voice was heard]’ (Terry).

For some, the experience of being listened to and making a contribution was an emotional one:

My oath! You know what I’ve done a bit of crying through this, when I got the job, when Bridget rang me up, Wow someone’s looking at ME for something. And when it finished, when the book [zine] came out, I started crying to my sister. I’ve achieved something. I’m 50 years old and I’ve done nothing with me life [until now]. Sam

Being heard and valued did not relate directly to being agreed with; not all group members believed that a print resource was the outcome they wanted from the group. Some were disappointed this was of more benefit to those who were new to the streets rather than the current homeless who ‘already know this stuff’ (Ash), but others saw this as a valid thing to do ‘Knowing there was very little information available to the homeless on the streets, it was way for me to give back, while I still had a clear mind’ (Jamie). But regardless of their feelings about the end‐product, group members unanimously said they believed they had been heard.

### 4.3 Capacity building: supporting people to participate safely, and in a way that is empowering

An initial task for the group members was to develop a Group Agreement (Appendix B) on the way they would treat each other during the workshops and ensure that it was safe, respectful and inclusive. This was done in an open forum with all members participating and having their ideas recorded on a whiteboard before they were developed into clear statements.

The Group Agreement was seen as a crucial element in providing guidance for the members about how to behave in a group, which for some was a new experience. One of the facilitators reflected that it took time to learn to listen and treat each other respectfully and referring back to the agreement was key to ensuring this happened.

Living on the streets in our own little individual cliques, or little groups we speak in, so we’re sorta used to, each one of us, we have a different set of rules or a different understanding of each other like not speaking over each other… Some things were a bit difficult, and probably did create a bit of disruption, but that’s our nature, some of us have been on the streets for 30 plus years. Chris

We did really well with it. The main thing was lots of us like to jump in…Needed to learn to let people fully speak, get their opinion out before we spoke. We all did really well, stuck to the agreement. Ash

Some group members were able to list very specific skills they had developed directly as a result of being part of the group, such as public speaking, or opportunities to be on other advisory committees. For most, the benefits they described were more around self‐confidence, social connectedness and making friends in an enjoyable setting.

A key thing the group members associated with being in the group was confidence, whether it was gaining or regaining confidence.

Able to talk a little bit more. More confident. Now understand more and bigger words… I could never be in a group, best working by myself, lose concentration too much in a group. Because we were all similar, similar experiences, it was easy to be in that group. If it was a group that was different, that wasn’t all homeless, I’d be out of whack. Ash

Probably I wouldn’t necessarily say new skills, I’d say refreshed my skills – as in talking to people… designing, coming up with ideas, even just writing stuff down I haven’t handwritten things for years. That type of skill was very useful. Jamie

The facilitators observed changes in the group members which they were not necessarily able to articulate themselves. In particular, they noted that with time group members became more comfortable with each other and with their role in the group. This manifested in them showing more empathy for other people, and also being better able to participate in group work through listening and allowing others to contribute. Developing empathy and the capacity to view things from the perspective of others allowed workshop members to develop productive and constructive responses that would benefit others. Additionally, some group members displayed more positivity and a sense of hopefulness as the group progressed.

### 4.4 Purposeful: working towards a useful outcome

In the first session, the purpose of a Working Group was made very clear, so that members understood it would be time‐limited and with a goal to achieve—in this case devising something which would help people to live more safely on the streets in the short term and help them to eventually link to services and supports over time that could help them avoid entrenched homelessness. The group took part in extensive activities such as reviewing the data from the surveys, developing Idea Matrices and discussions about realistic time frames and costs for an end‐product.

Some of the group members recalled that the group had always been set up to produce a printed resource of some kind: ‘a useful true fact pamphlet with 100% facts in that people need’ (Charlie) but others recalled the initial brief was much broader:

We were the voice of all homeless people, not just ourselves… to make something from a lived experience perspective rather than a worker perspective…wasn’t until about halfway through we came up with the idea of the helping out booklet. Alex

The decision to produce a zine/website/event disappointed some group members who would have preferred their ideas be taken up, which primarily revolved around strategies to improve access to storage and personal safety.

## 5 Discussion

This project demonstrated that it was possible to involve some of society's most vulnerable people in an extended co‐design project which was sensitively managed and appropriately resourced. This was a far more intense process than one‐off consultation or focus groups—which in some cases can be tokenistic—and given the difficult circumstances in which the members of the group lived, the on‐going engagement over an extended period of time by of the majority of the group is an achievement. Eight of the group members continued their involvement beyond the initial 12 Working Group sessions and transitioned into an Editorial Group which continues to oversee the production of the zine and website (which will be on‐going, subject to funding).

Critically, this project had significant resources. The project team combined street knowledge and experience in facilitating groups through a peer worker with a personal history which includes past lived experience of sleeping rough as well as a social worker with expertise in working with groups. They had the time and resources they needed to run this project. Funding was available to pay participants and to cover the direct costs of running the workshops. There was also a small amount of funding available to pay for design and development of an outcome of the co‐design process. The project was undertaken by an organization with the expertise and capacity to support it.

### 5.1 Essential elements in success

There were three key elements to successful application of the co‐design principles used in this project.

#### 5.1.1 Selecting appropriate group members

Inclusion is a core principle for successful co‐design; however, it can be difficult to attract marginalized groups to become involved in public participation groups.^8^ One suggestion is to recruit through established networks^7^; however, in this project, initial interest in participating in the Working Group was gauged from those who completed a survey, thus spreading the net well beyond known networks. The high level of interest then allowed the facilitators to use a detailed, multi‐step process to select group members.

The selection process went beyond choosing those with particular experiences or from specific demographics, or who had been involved in similar groups. The group was not restricted to those who had limited experiences living on the street or were generally coping well, they had extensive and varied histories of rough sleeping. The selection process allowed for people to be selected for their willingness and ability to commit to and contribute to the group. A diverse group of participants joined and stayed in the group.

#### 5.1.2 Making participation a positive experience

After recruiting the group, it was important to conduct it in a way which maintained interest, enthusiasm and on‐going participation. Every stage of this project was designed to ensure that the group members benefited in some way. Those who took part in the survey were reimbursed for their time, as well as being given other tangible items such as meal vouchers. More importantly, they were connected with services if it was appropriate. This kind of support goes beyond capacity building; it reflects the principles of the community health organization, which offers holistic care including referrals for diverse needs as they emerge.

The prime motivation described for being on the group was the desire to share insights and to help others who found themselves on the streets. A couple of group members described feeling overwhelmed and happy when it was confirmed they would be on the Working Group. Other studies have also described altruism as a motivator, but not with such a disadvantaged group.^22^ This group expressed a strong sense that their voices were being heard and valued, and for many, this level of recognition was a novel experience, reflecting the presence of the principle of equity and respect.

A crucial component in supporting safe participation was the agreement that was drawn up by the members, which clearly spelled out what was expected of them in their interactions within the group. They took total ownership of the agreement and saw it as a way to help manage both their own and others’ behaviour while in the group. Within each session, the group agreement was presented as a PowerPoint slide, and often read out, which served to remind people of how they had agreed to behave. Importantly, slides were also used to regularly restate the overall goal of the project as well as goals for specific meetings. These factors helped with setting boundaries and keeping the project on track. Some group members were keenly aware of their own difficulties and lack of experience in working in groups—as well as that of others’—and were grateful for the reminder about how to behave.

The less tangible aspects of being in the group were also crucial to the co‐design process. The members developed a strong sense of camaraderie as well as connections with the facilitators. They described feeling welcomed when they arrived, they chatted informally and shared information among themselves. The group was also run in a way which enabled members to withdraw if they were feeling upset or overwhelmed. They could have tea or coffee at any time or get a stress toy—which could also act as a signal to the facilitators that they may need some help. The facilitators used their experience as health professionals to monitor the health and well‐being of group members. They would debrief after each session and identify any concerns that could be addressed by referral or assertive outreach. Where there were concerns about group members’ well‐being, the project workers would follow‐up by phone between meetings. In addition, the meetings were deliberately conducted next door to a community health service, to encourage engagement and use the of health services available.

Several group members commented on the on‐going benefits of their experience in the project on their lives, reflecting achievement of the principle of capacity building that is empowering. The facilitators noted an increase in empathy, willingness to listen to others, and a more positive outlook by group members as the group progressed. One facilitator observed that planning tasks, keeping to a timeline, and being flexible are skills and abilities some group members would not have used much recently. Following their participation in the project, some of the group members were looking at participating in other committees or groups which would include a broader range of people, after gaining confidence and skills from this experience.

#### 5.1.3 Clarity of expectations at every stage

A crucial component the success of this project was clarifying expectations, both of what the group was trying to achieve and what was expected of the members, which was also spelled out in a consent form/contract‐style document outlining roles. This approach reinforced the principle of purposefulness; the group members were reminded of this at all stages of the project. From the first contact with potential group members, it was made clear that the commitment would be to 12 weekly group sessions and that the aim was to ‘reduce the health, legal and social impacts of sleeping rough’.

### 5.2 Outcomes of the co‐design process

The concrete outcome of this group was a three‐pronged information strategy (zine, website, event) of information that the working group identified as important, and of particular relevance to those new to the streets. It is estimated that at any time 50%‐60% of people in Melbourne are new to rough sleeping.^23^ There are numerous organizations which provide assistance for people experiencing homelessness, some funded by different levels of government and others by the not‐for‐profit sector. These services include accommodation support, material aid, legal and health services. A comprehensive 50‐page guide is produced annually, but it cannot be rapidly updated and is written in a way that is more appropriate for professionals than people on the streets. https://www.melbourne.vic.gov.au/community/health‐support‐services/social‐support/what‐we‐are‐doing/Pages/helping‐out.aspx.

As a result of the co‐design process, the group determined that there was a need for up‐to‐date information about where and how to access resources, facilities, and services, and how to live on the street in a way that minimizes harm. For example, the first issue presented information about ‘Stuff that can get you into strife when homeless in the city’, more formally known as the ‘Homelessness Protocol’ otherwise only available on a website. https://www.melbourne.vic.gov.au/sitecollectiondocuments/homelessness‐operating‐protocol.pdf The zine can help get information quickly to people and in a way which may resonate with them. The project team implemented an evaluation strategy to assess the reach and impact of the zine.

### 5.3 Challenges

#### 5.3.1 Young people

No young people (under 25) could be recruited to take part in the project. The facilitators made additional efforts to recruit younger people beyond the survey respondents, including making extra calls and approaching specialist youth services and workers. The facilitators felt young people were more accustomed to ‘drop‐in’ and casual engagement, and therefore were not able to commit to the 12‐week process. A separate youth‐focused co‐design may be necessary to reflect their particular experiences and issues.

#### 5.3.2 Withdrawals from the group

Three people left the working group before it concluded the co‐design process; one for health reasons and one because changes in personal circumstances that made participation difficult. They were all contacted for interviews for the evaluation but all declined, so they did not provide any feedback about their experiences. Choosing people who are living in such difficult personal circumstances makes some level of dropout almost inevitable. Rather than choosing people who were more stable, such as only those who were recently housed, the choice was made to include people with current or recent experience of sleeping rough because of the relevance of their knowledge and experience. The facilitators recruited a few more people than they thought were needed to allow for this natural attrition, as they did not want to damage the group dynamic by recruiting additional members at a later stage.

#### 5.3.3 Power differentials

Co‐design projects raise ethical issues^24^ and recruitment, maintaining engagement and managing power differentials are particularly challenging when projects involve vulnerable people.^8^ As described, the recruitment and running of this group was tailored to the needs of the participants, most of whom had not participated in anything similar previously. Power differentials can be hard to mitigate, and group members were aware that there was a management structure outside the group which would ultimately decide on the acceptability of their ideas. Every meeting included an update about what had occurred outside the group, so they felt included in all parts of the project. Issues with power differentials were also reduced by having a group in which the majority of people were experiencing homelessness or had done so recently. The lived experience of the peer worker and the other facilitator's experience as a social worker sensitized them to issues of power, and there were no other professionals or staff from cohealth participating in the group. This assisted group members to relate to each other, unlike some co‐design in health‐care settings where groups may include medical staff and patients. Also, the group was tasked with creating a new solution, rather than improving an existing one; therefore, they were not in the position of directly criticizing cohealth.

#### 5.3.4 COVID‐19

The advent of COVID‐19 meant that distribution plans for the zine needed to be adapted, as some services shut down, and many rough sleepers were moved temporarily into hotels or other accommodation. The group determined alternative strategies for distribution such as including copies in essential item packages distributed to people on the streets and outreach teams from the community health organization to taking the zines to hotels. The content was also rapidly modified to include a supplement about COVID‐19, demonstrating the value of having on‐going mechanisms for working with vulnerable populations in crises, as recently suggested.^25^

Meetings of the group were also impossible during a hard lockdown in Melbourne, and issues around the digital divide are very prominent in this group.^26^ Although most people who are homeless have basic mobile phones, few will have access to software or skills to participate in online meeting platforms. Thus, contact became predominantly one‐on‐one between a facilitator and group member.

## 6 CONCLUSIONS

This project demonstrated that very vulnerable and disadvantaged people were willing to take part in a co‐design process that involved multiple group meetings over more than 3 months, and that they welcomed the opportunity to have their voices heard in a safe setting and their ideas put into action. The selection of the group members and the creation of an environment where they felt valued and nurtured was crucial to allowing them to share their voices and actively create practical resources. As one person summed up: ‘No‐one has really listened to my side before, anything I’ve got to say before. Now I’ve done this’.

## CONFLICT OF INTEREST

The authors declare no conflict of interest.

## Data Availability

Research data are not shared due to privacy considerations

## APPENDIX A

### INTERVIEW SCHEDULE

#### Recruitment

When you were first asked if you were interested in being part of the working group, what made you think it was something you would like to do*? (probe: being paid, interested in giving opinions/thought they had something to offer/something to do….)*

After you said you were interested, what happened before you were selected to be part the group?

Would you make any changes to the way you were asked if you would like to participate in the working group, or the selection you went through?

#### Meetings

Can you describe the purpose of the Working Group? *Probe: partners, equal,*

Can you describe **your** role in the Working Group? Do you feel like your voice was heard? Were the voices of everyone on the working group heard? Is there any way you feel this could be improved?

What were the main things that the Group Agreement emphasized? Was this effective?

Do you feel you were given enough support to participate in the weekly sessions? *Probe material support, emotional support*

#### Resource

There were many ideas that came from the survey. How did you feel about the way decisions were made about what was discussed at the Working Group and went into the resource?

Are you happy with the resource that was produced? Do you feel you had a genuine role in producing it?

#### Overview

Do you think you developed new skills or had new opportunities as a result of being on the WG?

If cohealth, or a similar organization, was going to set up another co‐design project like this, what do you think they should do the same way? What could they do differently which would make it better?

Any other comments?

## APPENDIX B

### ROUGH SLEEPING CO‐DESIGN PROJECT—WORKING GROUP

#### Group Agreement

**Give each other space—**To talk or to be quiet, whatever they might need.

**Be empathetic—**Show this in your language and interactions.

**Respect our stories and other survey participants’ stories**

**Everyone's got a voice and freedom of speech *but this doesn't excuse racism, sexism, homophobia, transphobia or other types of discrimination***.

**Think about your tone when you are speaking—**try to change it if it sounds judgemental, condescending, attacking, etc

**Help each other out—**We all have our struggles and strengths. We all have good days and bad days.

**Be open minded—**Show this in your language and interactions.

**Stop yourself from speaking over others or cutting others off—**Let people finish or wait for a pause

#### Listen to others

**Confidentiality—**Don't discuss others stories outside of the group, don't discuss peoples full names outside of the group unless they give you consent for this.

## References

[1] Sanders EBN , Stappers PJ . Co‐creation and the new landscapes of design. CoDesign. 2008;4(1):5‐18.

[2] Blomkamp E . The promise of co‐design for public policy. Aust J Public Admin. 2018;77(4):729‐743.

[3] Smith JA , Judd J , Bainbridge R , et al. Are we going “co‐crazy”? An opportunity to learn from health promotion foundations. Health Promot J Austr. 2018;29(3):223‐224. 30511493 10.1002/hpja.215

[4] Bombard Y , Baker GR , Orlando E , et al. Engaging patients to improve quality of care: a systematic review. (Report). Implement Sci. 2018;13(1):98. 30045735 10.1186/s13012-018-0784-z PMC6060529

[5] Steen M , Manschot M , Koning N . Benefits of co‐design in service design projects. Int J Design. 2011;5(2):n/a.

[6] Bonevski B , Randell M , Paul C , et al. Reaching the hard‐to‐reach: a systematic review of strategies for improving health and medical research with socially disadvantaged groups. BMC Med Res Methodol. 2014;14(1):42. 24669751 10.1186/1471-2288-14-42 PMC3974746

[7] Domecq JP , Prutsky G , Elraiyah T , et al. Patient engagement in research: a systematic review. BMC Health Serv Res. 2014;14(1):89. 24568690 10.1186/1472-6963-14-89 PMC3938901

[8] Mulvale G , Moll S , Miatello A , et al. Codesigning health and other public services with vulnerable and disadvantaged populations: Insights from an international collaboration. Health Expect. 2019;22(3):284‐297. 30604580 10.1111/hex.12864 PMC6543156

[9] de Freitas C , Martin G . Inclusive public participation in health: Policy, practice and theoretical contributions to promote the involvement of marginalised groups in healthcare. Soc Sci Med. 2015;135:31‐39. 25939074 10.1016/j.socscimed.2015.04.019

[10] Wood L , Flatau P , Zaretzky K , Foster S , Vallesi S , Miscenko D . What are the health, social and economic benefits of providing public housing and support to formerly homeless people? Vol. AHURI Final Report No. 265. 2016. https://www.ahuri.edu.au/research/final‐reports/265. Accessed January 31, 2021.

[11] Clarke A , Parsell C , Vorsina M . The role of housing policy in perpetuating conditional forms of homelessness support in the era of housing first: evidence from Australia. Hous Stud. 2020;35(5):954‐975.

[12] Australian Bureau of Statistics . 2049.0 ‐ Census of Population and Housing: Estimating homelessness, 2016. Canberra: ABS; 2018.

[13] Fazel S , Geddes JR , Kushel M . The health of homeless people in high‐income countries: descriptive epidemiology, health consequences, and clinical and policy recommendations. Lancet. 2014;384(9953):1529‐1540. 25390578 10.1016/S0140-6736(14)61132-6 PMC4520328

[14] Davies A , Wood LJ . Homeless health care: meeting the challenges of providing primary care. Med J Aust. 2018;209(5):230. 30157413 10.5694/mja17.01264

[15] Luchenski S , Edge C , Hutchinson‐Pascal N , et al. Co‐production of a research and advocacy agenda for Inclusion Health. Lancet. 2019;394:S68.

[16] O'Reilly F , Doyle J , Keenan E . Innovative co‐design of integrated services designed to improve access to health care. Int J Integr Care. 2017;17(5):480.

[17] Chiappalone S , Kelly B , Mullins R . Minimising the harm of rough sleeping: Integrating lived experience into addressing the health, legal and social risks of primary homelessness. Parity. 2020;33(2):17‐19.

[18] Roper C , Grey F , Cadogan CE . Co‐production: putting principles into practice in mental health contexts. 2018. https://healthsciences.unimelb.edu.au/__data/assets/pdf_file/0007/3392215/Coproduction_putting‐principles‐into‐practice.pdf. Accessed January 31, 2021.

[19] Co‐design, Initiative . Shared perspectives on authentic co‐design: putting consumers and carers at the centre of mental Health reform. 2016. https://auspwn.files.wordpress.com/2016/05/codesign‐shared‐perspectives‐report‐vf1‐5‐040616.pdf. Accessed January 31, 2021.

[20] Braun V , Clarke V . Using thematic analysis in psychology. Qual Res Psychol. 2006;3(2):77‐101.

[21] Thorne S . Verbatim quotations in qualitative research reports on the use and abuse of verbatim quotations in qualitative research reports. Nurse Author Ed. 2020;30:4‐6.

[22] Hemphill R , Forsythe LP , Heckert AL , et al. What motivates patients and caregivers to engage in health research and how engagement affects their lives: qualitative survey findings. Health Expect. 2020;23(2):328‐336. 31800154 10.1111/hex.12979 PMC7104645

[23] Nicholson T . Rough Sleeping in Victoria: Situation Appraisal. 2017. https://chp.org.au/wp‐content/uploads/2017/06/Rough‐sleeping‐in‐Victoria‐Situation‐appraisal.pdf. Accessed January 31, 2021.

[24] Kelly J . Towards ethical principles for participatory design practice. CoDesign. 2019;15(4):329‐344.

[25] Marston C , Renedo A , Miles S . Community participation is crucial in a pandemic. Lancet. 2020;395(10238):1676‐1678. 32380042 10.1016/S0140-6736(20)31054-0 PMC7198202

[26] Chew‐Graham CA . Involving patients in research during a pandemic. Health Expect. 2020;23(3):521‐522. 32594658 10.1111/hex.13079 PMC7321720

